# Theranostics in immuno-oncology using nanobody derivatives

**DOI:** 10.7150/thno.34941

**Published:** 2019-10-15

**Authors:** Quentin Lecocq, Yannick De Vlaeminck, Heleen Hanssens, Matthias D'Huyvetter, Geert Raes, Cleo Goyvaerts, Marleen Keyaerts, Nick Devoogdt, Karine Breckpot

**Affiliations:** 1 Laboratory for Molecular and Cellular Therapy (LMCT), Vrije Universiteit Brussel (VUB), Laarbeeklaan 103, B-1090 Brussels; 2 In Vivo Cellular and Molecular Imaging Laboratory (ICMI), VUB, Laarbeeklaan 103, B-1090 Brussels; 3 Unit of Cellular and Molecular Immunology (CMIM), VUB, Pleinlaan 2, B-1050 Brussels; 4 Myeloid Cell Immunology Lab, VIB Inflammation Research Center, Pleinlaan 2, B-1050 Brussels, Belgium; 5 Nuclear Medicine Department, UZ Brussel, Laarbeeklaan 101, B-1090 Brussels.

**Keywords:** single domain antibody, nanobody, cancer, molecular imaging, immunotherapy

## Abstract

Targeted therapy and immunotherapy have become mainstream in cancer treatment. However, only patient subsets benefit from these expensive therapies, and often responses are short‐lived or coincide with side effects. A growing modality in precision oncology is the development of theranostics, as this enables patient selection, treatment and monitoring. In this approach, labeled compounds and an imaging technology are used to diagnose patients and select the best treatment option, whereas for therapy, related compounds are used to target cancer cells or the tumor stroma. In this context, nanobodies and nanobody-directed therapeutics have gained interest. This interest stems from their high antigen specificity, small size, ease of labeling and engineering, allowing specific imaging and design of therapies targeting antigens on tumor cells, immune cells as well as proteins in the tumor environment. This review provides a comprehensive overview on the state-of-the-art regarding the use of nanobodies as theranostics, and their importance in the emerging field of personalized medicine.

## Introduction

The description of camelid single domain antibodies (sdAbs), as we know them today, was preceded by a report published by *Ward S.* in 1989. In this report, the binding characteristics of isolated variable domains (V_H_) from the heavy chain of antibodies, generated after immunizing mice with either lysozyme or keyhole-limpet hemocyanin, was described 1. The V_H_ genes were expressed in *E. coli* and the V_H_ were characterized by nanomolar affinity for their target. However, the antigen-binding affinity, stability and solubility of the V_H_ were lower than those of the parent antibody, posing major challenges for commercial application. It was not until 1993 that *Hamers R et al.* described heavy-chain-only antibodies (HCAbs) in camelids, from which high affinity, functional camelid sdAbs are derived 2.

*Hamers R.* and his team from the Free University Brussels (Vrije Universiteit Brussel, VUB [Dutch]) analyzed serum samples from dromedaries (Arabian camel) and discovered the presence of immunoglobulins (IgGs) lacking a light chain. These HCAbs have a molecular weight of ~90 kDa and contributed up to 75% of all serum IgGs. Also other members of the *Camelidae* family were shown to possess HCAbs with concentrations varying between 30-50%. Blotting experiments and radioimmunoprecipitation were used to show the high affinity of HCAbs. The antigen binding part of HCAbs was confined to one single domain, known as the variable domain of the heavy chain of the HCAb (V_H_H). *Ghahroudi et al.* were the first to show that camelid sdAbs are well expressed in *E. coli*, and are highly stable and soluble, making them interesting for commercial applications 3. Thus begins the exploratory phase for the development of camelid sdAbs that resulted in the foundation of Ablynx^®^ in 2001, as a spin-off company from the VUB and the Flemish Institute of Biotechnology (Vlaams Instituut voor Biotechnology, VIB [Dutch]), that refers to V_H_H and camelid sdAbs as nanobodies™.

**Figure 1:**
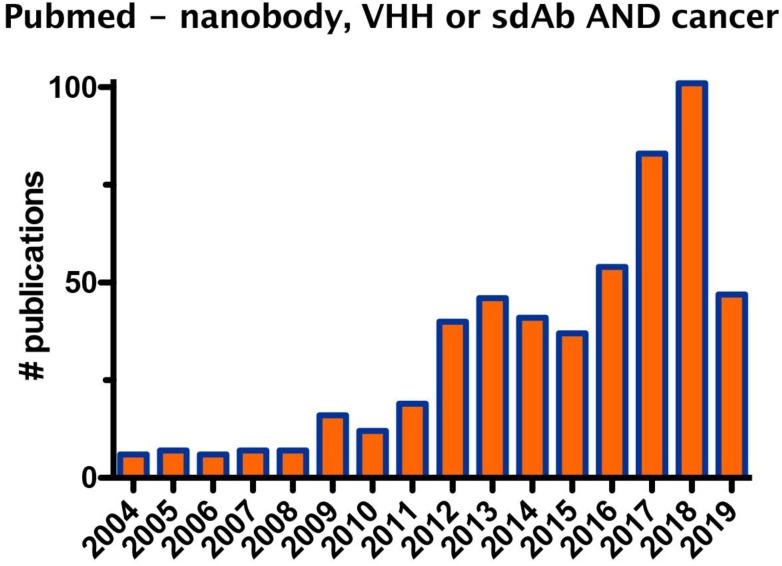
Graph showing the number of publications on the description of nanobodies in oncology during the period from 2004 to 2019.

Since their discovery, nanobodies received increasing attention, as exemplified by the growing number of studies that evaluate the application of nanobodies in the fields of biotechnology and medicine, in particular for oncology, the focus of this review (Figure 1).

The developments in nanobody discovery together with the many advantageous properties of nanobodies led to their exploration in clinical trials conducted by large biopharmaceutical companies 4. Meanwhile, main patent claims on nanobodies are expiring, which will fuel the growing interest in commercializing nanobodies as research, therapy and diagnostic agents. Following the description of the structure and merits of nanobodies, we will address the different diagnostic and therapeutic applications in the context of immuno-oncology in more detail.

## Nanobody structure and development platforms

**Figure 2:**
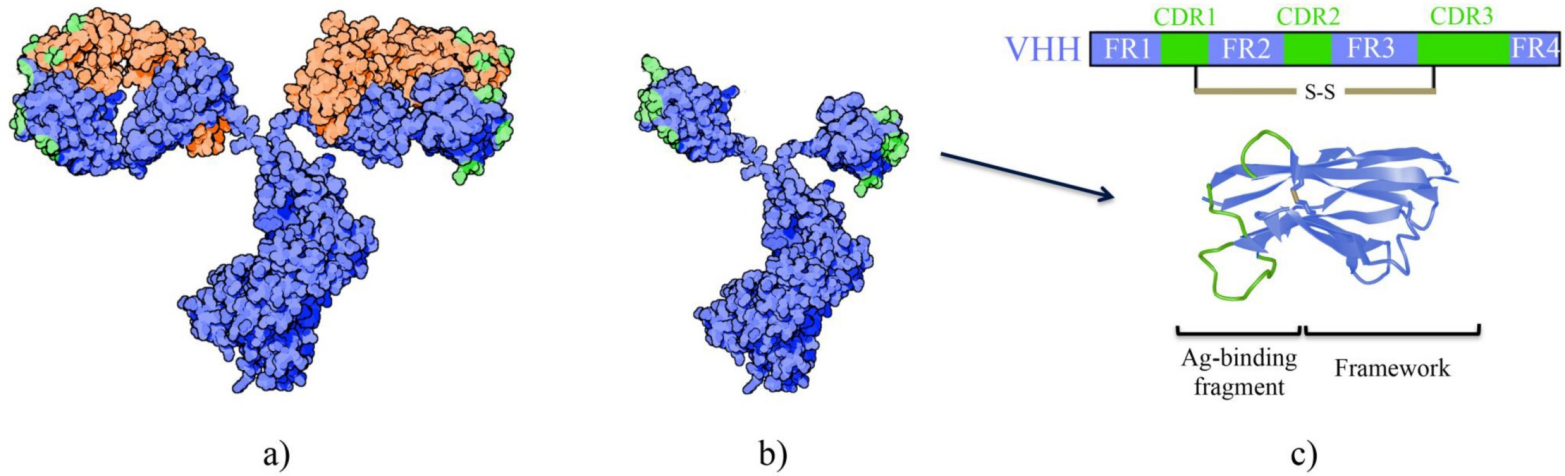
A schematic representation of the differences between a conventional antibody (a) and a HCAb (b). The antigen-binding domain from the HCAb is referred to as a V_H_H, nanobody or sdAb (c).

HCAbs are homodimers consisting of two heavy chains linked with a disulfide bond. These heavy chains are comprised of a variable (V_H_H) and a constant region however lack the C_H_1 domain found in conventional antibodies 5. Three complementarity determining regions (CDRs) form the antigen binding site of HCAbs, as opposed to six CDRs found in conventional antibodies that also contain light chains (Figure 2). The solvent-exposed framework region 2 (FR2) in V_H_H is more hydrophilic than the corresponding V_H_ fragment in conventional antibodies 6. The distinctive CDR3 loop in V_H_H plays a crucial role in antigen binding. Especially in camels and dromedaries, the single domain can have a particularly long and diverse CDR3 that is the result of recombination of different V-D-J germline elements, junctional diversity and hypermutation, and selection during *in vivo* maturation of more functional and soluble nanobodies with a long CDR3 7. Frequently the long CDR3 extends out and allows high affinity binding to a concave epitope at active sites of proteins that are usually inaccessible to antibodies 8-10. Moreover, besides CDR3, also CDR1 and CDR2 contribute to target binding, involving more hydrophobic amino acids in their paratope, and a surprisingly high amount of residues in framework regions make contacts with the antigen. It is suggested that the interaction of nanobodies to their targets are more similar to general protein-to-protein interactions instead of antibody-to-antigen interactions 10. Other differences to conventional antibodies have evolved to ensure large repertoire diversity and high binding capacity in the absence of light chains and include (1) an extended CDR1 region towards the N-terminal end, (2) involvement of FR2 in shaping the CDR3 loop and (3) extensive somatic hypermutation 11. Finally, disulfide bonds present in the V_H_H, especially those derived from camel and dromedary, confer extra stability 12.

The generation of a V_H_H library against an antigen of interest has already been described in numerous publications. The vast majority of isolated nanobodies described to date are isolated using the same procedure, namely selections of phage libraries displaying V_H_H retrieved from immunized camelids 13. In short, an animal from the *Camelidae* family like an alpaca or a dromedary is immunized with a source of antigen (frequently recombinant protein). Approximately 40 days later, peripheral blood lymphocytes are isolated and subsequent isolation of RNA is performed. The V_H_H gene fragments are amplified using a PCR and cloned in a phagemid vector to an *in vivo* matured V_H_H library. The library is phage-displayed and subjected to several consecutive rounds of biopanning on solid phase coated recombinant target protein or on cells, enriching antigen-specific phages with each round. Recently, newer techniques have been reported that allow improved screening of nanobody immune libraries using yeast surface display platforms or genetically encoded barcoding peptides 14-16. Finally, positive clones are cloned in an appropriate expression vector allowing nanobody production in microbial hosts like *E. coli*, *S. cerevisiae* or *P. pastoris* 17-19, in mammalian cells and plants 20. Bacteria produce the nanobodies in their cytosol or in periplasm. Nanobody extraction from the periplasm is performed through osmotic shock, while lysis using sonication or by freeze-thaw cycles is necessary for nanobody extraction from the cytosol. Yeast and mammalian cells secrete nanobodies at high yields in the culture supernatant, guaranteeing correct post-translational modifications like the formation of disulfide bonds, but care should be taken to avoid nanobody glycosylation as this can have a negative impact on its functionality and *in vivo* behavior 20.

The classic acquisition of a V_H_H library by immunizing *camelidae* is straightforward but inconvenient from the point of view of animal protection and costs to maintain these large animals. Transgenic mice that express HCAbs by their B cells were generated by Janssens* et al.* and could serve as an alternative host for immunization 21. This transgenic mouse was realized by recombining two llama variable V regions and the human D, J, Cμ and/or Cγ constant regions to generate a hybrid llama/human antibody locus. Recently, efforts has been made to develop *in vitro* selection of nanobodies against a target protein by for example cDNA display methods with synthetic or semi-synthetic libraries 22-25. Although some studies reported this method for selection of nanobodies against their target of interest, it must be mentioned that affinities were usually lower, in the range of 10^-7^ to 10^-8^ M, and that this approach demands large library sizes and sophisticated selection procedures, which currently is prohibitive for widespread implementation 26-28.

## Advantages and disadvantages of nanobodies

A key characteristic of nanobodies is their small size. Nanobodies are 2.5 nm in diameter, 4 nm in height with a molecular mass of around 12-15 kDa. Other advantageous traits include (1) high affinity, (2) high specificity, (3) low off-target accumulation, (4) high (thermo)stability and (5) good solubility 29. Due to their small size, nanobodies have the distinct feature of penetrating dense tissues like tumors very well 30. Additionally, some nanobodies have been described to even transmigrate through the brain endothelial cell layer 31-33. Moreover, nanobodies allow targeting hidden epitopes of certain proteins and at locations difficult to approach by conventional antibodies, which include ion channels 34, G protein-coupled receptors 35,36 and immune synapses 37. Ease of molecular engineering, low immunogenicity, and ease and relative low cost of production are other advantages that make nanobodies interesting compounds for various immuno-oncology applications 38.

For certain therapeutic applications, maximal delivery of the nanobody in the cancer lesion is desired, which is a disadvantage of these fast-clearing proteins. Several strategies can be explored to enhance the delivery of nanobodies to the tumor site. One of these is extending the serum half-life of the nanobody using different formatting options. Examples hereof are fusion of the nanobody directly with albumin, polyethylene glycol (PEG; PEGylation) or IgG-Fc (allowing recycling through the neonatal Fc receptor [FcR]), or indirectly with a second nanobody that binds albumin or the neonatal FcR, thereby generating bispecific constructs 39-42. Another option to obtain maximal delivery in the tumor site is to use a gene therapy approach, which could be highly feasible for nanobodies as these are simple proteins expressed from one single gene 43,44. A continuous, local secretion of nanobodies by genetically engineered cells in the tumor microenvironment (TME) will maximize their delivery to the target of interest and will moreover minimize the risks associated with systemic distribution. Additionally, nanobodies are well suited for cytosolic expression due to their ability to fold in reducing environments like the cytosol, a feature that has been widely exploited for *in cellulo* tracking of intracellular proteins via microscopic imaging 45,46.

As a result of their easy cloning, nanobodies have also been explored for other gene-based immuno-oncology approaches, as exemplified by the use of nanobodies to design (1) vaccines targeted to antigen-presenting cell (APC) subsets or (2) chimeric antigen receptors (CARs) to direct the specificity of cancer killing cells 47-57. The ease of molecular engineering is also exploited to generate multi-specific nanobody formats purposed to engage cancer killing immune cells, in particular cytotoxic T lymphocytes (CTLs) and natural killer (NK) cells. An example hereof is the development of so-called bispecific light T cell engagers (LiTEs) in which scFvs targeting the CD3 molecule on T cells are coupled to nanobodies targeting EGFR tumor on cancer cells. Consequently a bridge is formed between cancer cells and T cells and these T cells are activated through cross-linking of the CD3 molecule 58,59. The ability to design multi-specific and chimeric nanobody formats is furthermore exploited to generate so-called bispecific killer cell engagers (BiKEs), nanobody-based cancer therapy agents that engage NK cells to exert antibody-dependent cell-mediated cytotoxicity (ADCC) 60,61. ADCC is triggered when Fc is bound by FcRs on NK cells. The lack of an Fc in nanobodies implies that nanobodies in some cases might exhibit lower therapeutic potency compared to antibodies. To circumvent this, nanobodies targeting the FcγRIII have been developed 62. Coupling of this nanobody to an anti-carcinoembryonic antigen (CEA) specific nanobody resulted in potent killing of CEA^+^ cells 63. Consequently, combining an FcR-specific nanobody with a nanobody binding to a target of interest could be conceived when Fc effector functions are required. As these multi-specific nanobodies are still relatively small in size, they retain a good tumor penetration capacity.

Another potential disadvantage of nanobodies is that the human immune system can potentially perceive them as foreign, as they are derived from camelid HCAbs. Nonetheless, nanobodies are considered to be weakly immunogenic due to their high similarity with human V_H_ fragments and their properties, including size, monomeric form, solubility and lack of an Fc fragment 64. Furthermore, it is suggested that the immunogenicity of nanobodies could be minimized by humanization 65,66. Nanobody humanization is generally performed by surface veneering into residues that are encoded in the human germline. The change of each individual residue replacement, in particular in FR2, should be carefully monitored as this can influence both nanobody functionality and, importantly, its solubility which is an important trigger of immunogenicity 65,67. The necessity to humanize the nanobody sequence is further questioned by the observation that anti-drug immune responses against a humanized tetravalent nanobody targeting death receptor (DR) 5 on cancer cells, were observed and were the main reason to stop clinical evaluation 68. Some studies support the notion that nanobodies and even V_H_ fragments can be immunogenic 68-70, while other studies with other nanobody constructs showed no signs of immunogenicity 71-73. To date, there is a lack of understanding on the immunogenicity of non-humanized and humanized nanobodies due to insufficient data. Therefore, studies on this subject are warranted to demonstrate the possible limitations on the extent of manipulating nanobodies. As nanobodies are increasingly used in clinical studies, data will become available to address this question. Meanwhile, several companies focus on development of nanobodies and their use for diagnosis and/or therapy. Examples hereof are Ablynx^®^, Novamab, NanoMab, Orionis Biosciences, Helix Biopharma, Nanjing Legend Biotech and Camel-IDS, which have partnered with leading pharmaceutical companies to develop nanobodies among others to tackle cancer. Of these companies, Ablynx^®^ recently brought an anti-von Willebrand factor-specific nanobody to the market for the treatment of thrombotic thrombocytopenic purpura, highlighting the potential of nanobodies as innovative medicines 74.

## Nanobodies represent a versatile platform for imaging of cancer cells and their environment

The role of immune cells in shaping a tumor and in influencing the outcome of cancer therapy is generally recognized 75,76. As a consequence when diagnosing cancer, one would like to know as much as possible about the tumor, such as the presence of targetable tumor antigens and the immune contexture, to plan and monitor the most effective treatment. This can be achieved via molecular imaging, the use of labeled indicator molecules that can be imaged in a noninvasive manner. Nanobodies are attractive tools for this purpose 77.

### Modalities for nanobody-based imaging

For noninvasive imaging, nanobodies need to be labeled with an imaging probe that can consist of a radioisotope, fluorescent dye, microbubble or a chemical like gadolinium, allowing imaging via technologies such as single photon emission computed tomography (SPECT), positron emission tomography (PET), optical imaging (OI), ultrasound (US) and magnetic resonance imaging (MRI) 30. While these different imaging modalities exist, the majority of nanobody-mediated imaging studies use SPECT and PET, because these radioisotope-based techniques have a high sensitivity and offer quantitative information 78-80. In preclinical studies, nanobodies often contain a genetically inserted C-terminal hexahistidine tag for purification purposes, which can be complexed with ^99m^Tc(CO_3_), a γ-emitting radionuclide that is easily detectable using SPECT 81. For PET, which is clinically more relevant, nanobodies are labeled with positron-emitting radionuclides, exemplified by ^18^F, ^64^Cu, ^68^Ga and ^89^Zr. Frequently used radioisotopes are ^18^F and ^68^Ga because of their short half-life, 68 and 110 minutes respectively, matching the biological half-life of nanobodies when injected intravenously 82. For coupling of these radioisotopes to nanobodies site-specific labeling is desired to obtain homogenous and consistent tracers. A good example of site-specific conjugation is the transpeptidase sortase A-mediated ligation. The latter catalyzes the formation of a peptide bond between the C-terminally expressed LPXTG peptide motif of the nanobody and the N-terminal oligo-glycine motif on the label 83,84. Another example is the conjugation of maleimide-functionalized labels on an unpaired cysteine on the nanobody 85-87. An alternative to radiolabeling of nanobodies is the use of fluorescent dyes that can be combined with OI. For *in vivo* imaging, near-infrared (NIR) emitting fluorophores are the label of choice, as these provide strong contrast and resolution combined with signal detection in depths ranging from several hundred µm to one cm 88. Examples of NIR fluorophores include IRDye-680RD or -800CW, Cy5 and AlexaFluor 680 77. Advantages of OI are its flexibility, simplicity and cost-effective character, as in contrast to radioisotope-mediated imaging, it does not require dedicated facilities. This imaging modality is often used to study surface lesions during surgical or endoscopic procedures, as OI dyes have limited tissue penetrating capacity compared to radioisotope-based imaging. The use of US has been studied as an alternative to radiolabeled nanobodies while retaining the ability for high-resolution images 89. This type of imaging requires conjugation of nanobodies to US contrast agents, microbubbles or nanobubbles that after intravenous administration allow the molecular characterization of the vascular wall 90,91. Finally, *Prantner et al.* evaluated cross-reactive nanobodies targeting mesothelin-expressing ovarian cancer for MRI imaging 92. Their nanobody-coated superparamagnetic nanoparticles allowed mesothelin-detection in xenografted tumors using MRI.

### Imaging of cancer cells

**Figure 3:**
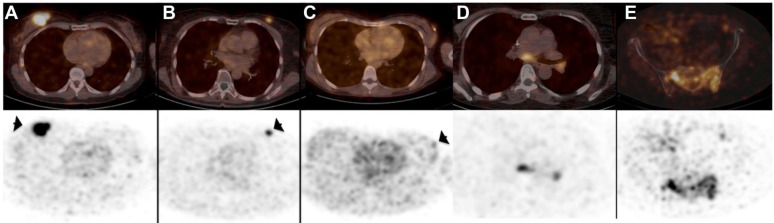
PET/CT scans (top) and PET scans (bottom) after injection of ^68^Ga-HER2-Nanobody showing uptake in primary breast carcinoma lesions (arrows) (A-C) and metastatic lesions in lymph nodes in mediastinum and left hilar region (D) and bone metastasis in pelvis (E). Adapted with permission from 73, copyright 2016.

Nanobody-based imaging has been extensively studied for detection of cancer cells themselves, mostly in preclinical studies. Antigens that have been targeted include but are not limited to CEA, epidermal growth factor receptor (EGFR), HER2, prostate-specific membrane antigen (PSMA), CD20 and CD38, as reviewed by *Debie et al.* 77. With regard to clinical testing the most advanced nanobody-based imaging agent is the ^68^Ga-coupled anti-HER2 nanobody 2Rs15d for PET imaging of breast cancer patients 93. Results of the first clinical trial with nanobody 2Rs15d were published in 2016 and showed detection and imaging of HER2 in the primary tumor as well as local or distant metastases as soon as 60 minutes post-injection without any adverse effects, such as renal toxicity and tracer-induced antibodies 73. The detection of HER2 using this nanobody was shown to be highly specific reaching SUV values of 11.8 for the primary lesions and 6.0 for metastases (Figure 3). Moreover, background uptake was very low with the exception of signals observed in the kidneys, intestines and liver. Recently, a phase II clinical trial evaluating the potential of ^68^Ga-NOTA-2Rs15d to detect brain metastasis has been initiated (NCT03924466). Implementation of nanobody-based imaging of cancer markers can be a guide for therapy selection, in particular as targeted therapies have been developed for many of these cancer markers, some of which are based on the use of nanobodies, as exemplified by the use of anti-CD20 and anti-HER2 nanobodies for targeted therapy 94-96.

### Imaging the tumor stroma

Solid tumors can be considered as abnormal organs comprising multiple immune cell types, new blood vessels and extracellular matrix (ECM). As cancer cells intimately interact with their surroundings and as this crosstalk often promotes tumor progression and even therapy resistance, it is of interest to visualize (and target) these components as well. Recently, the generation of diverse nanobody libraries against ECM proteins associated with cancer was described 97. From these libraries, a nanobody that targets the alternatively spliced EIIIB domain of fibronectin, an important component of the ECM and neovasculature, was used to show the broad applicability of ECM-targeted nanobodies to image tumors and metastases using two-photon immuno-fluorescence and noninvasive immuno-PET/CT imaging 98. In line with this topic is the visualization of newly forming blood vessels. As endothelial cells that line blood vessels are genetically more stable than cancer cells, they have been proposed as targets for anti-angiogenic therapy 99. Visualization of tumor endothelial cells that express vascular cell adhesion molecule-1 (VCAM-1) has been described using anti-VCAM-1 nanobody decorated microbubbles 91, and also radiotracer variants of this nanobody are available 100,101. This is important as a correlation between VCAM-1 expression on endothelial cells and vessel density as well as metastasis was reported 102,103. Moreover, VCAM-1 expression on cancer cells has been linked to immune evasion 104. It was shown using the TC-1 lung epithelial cancer model that tumors with high VCAM-1 expression were devoid of CD8^+^ tumor-specific T cells 105. These studies suggest that VCAM-1 nanobodies could provide valuable information on the resistance of tumors to immune-mediated killing when combined with nanobodies generated to image CD8^+^ T cells. Such nanobodies have been developed by *Rashidian et al*. and were studied as a means to predict response to anti-cytotoxic T lymphocyte antigen-4 (CTLA-4) immune checkpoint (ICP) therapy in a mouse melanoma model 106.

As blockade of inhibitory ICPs, such as CTLA-4 and programmed death-1 (PD-1): programmed death-ligand 1 (PD-L1), receives increasing attention in immuno-oncology, nanobody-based probes to image the expression of these ICPs have been developed. PET/CT imaging of naive and tumor bearing mice was performed using the ^18^F or ^89^Zr radiolabeled anti-CTLA-4 nanobody H11 107*.* Nanobodies to image the expression of PD-L1 in the TME have also been developed 108. High contrast images showing mouse PD-L1 expression in syngeneic mouse tumors were obtained 1 hour after inoculation of a ^99m^Tc-labeled nanobody using SPECT 109. Moreover, human PD-L1 specific nanobodies were generated for SPECT 110,111 and PET imaging 112. Regarding SPECT imaging, the nanobody K2 demonstrated several interesting properties, among which high specificity and affinity for human PD-L1, low kidney retention and competition with the FDA approved anti-PD-L1 antibody, avelumab 111. For PET imaging, ^89^Zr labeled KN035, a bivalent nanobody containing an Fc tail, was able to monitor PD-L1 levels using PET in nude mice bearing human xenografts 112. Additionally, a first-in-human study demonstrated that SPECT imaging with nanobody ^99m^Tc-NM-01 is safe and suited to evaluate PD-L1 levels at the tumor site as soon as 2 hours after injection 110.

Imaging of proteins such as CD8, VCAM-1, CTLA-4 and PD-L1 provide a first glance on the immune contexture and possibly immune resistance of tumors. However, certain immune cell types like macrophages have been designated as culprits in the development of tumors and in the failure of many immunotherapies 75. Macrophages in the TME can present themselves in many forms, and efforts have been placed in identifying markers that can discriminate pro- from antitumor macrophages 113. It was reported that macrophage mannose receptor (MMR, CD206) is highly expressed on tumor-promoting macrophages. Nanobodies to detect MMR have been developed and were shown to bind MMR^+^ macrophages in hypoxic regions. These ^99m^Tc-labeled anti-MMR nanobodies were also used to image MMR levels in tumors using SPECT/CT 114.

A comparison of this nanobody labeled with ^99m^Tc versus ^18^F showed that labeling with ^18^F reduced both liver and kidney uptake 115. The first steps towards clinical translation of this approach using a ^68^Ga-NOTA-anti-MMR nanobody for PET/CT imaging were recently taken 116. In contrast to MMR, expression of MHC-II on macrophages (and dendritic cells [DCs]) is considered a good prognostic marker, as it is associated with antigen presentation to CD4^+^ T cells. An MHC-II targeting nanobody that was labeled with ^64^Cu was able to image MHC-II^+^ cells in the spleen and bones of NOD/SCID humanized mice using PET/CT 117. Further, detection of 1 mm-sized tumors with a high affinity ^18^F-labeled anti-MHC-II nanobody was shown to be more accurate than with ^18^F-FDG 118. Other probes that were developed to image (and target) APCs, include the probes DC1.8 and DC2.1 119. DC2.1 was shown to recognize a wide range of myeloid cells, while DC1.8 only bound immature DCs. This binding pattern was reflected in their biodistribution when labeled with ^99m^Tc, with DC2.1 showing accumulation in liver, spleen and lungs, and DC1.8 primarily showing signals in skin. With the growing interest in targeting tumor-associated DCs to unleash an antitumor immune response, further studies on the potential of DC-targeting nanobodies to image specific DC subsets is warranted 120.

## Therapeutic application of nanobodies and nanobody derivatives in immuno-oncology

Surgery, chemotherapy and radiotherapy have long been considered the best options for cancer treatment. These therapies are an indiscriminate warfare, coinciding with damaging side effects and failing to protect against recurring cancer cells. Our growing understanding on how cancer develops has opened new avenues to treat cancer. Cancer is driven by corrupted messages from our own genes, caused and influenced by a range of factors, not in the least our own immune system. The identification of molecular accelerators of cancer cells, such as HER2, led to the development of molecularly targeted treatments, designed to bind and override faulty molecules in cancer cells. Moreover, the identification of immune cells and immune pathways acting as a brake or accelerator on cancer cells, led to the idea of manipulating the immune system to fight cancer. In particular the latter has gained increasing interest, as with immunotherapy comes the promise of a systemic treatment leading to a long-lasting immunological memory capable of tackling recurrent cancer cells.

**Figure 4:**
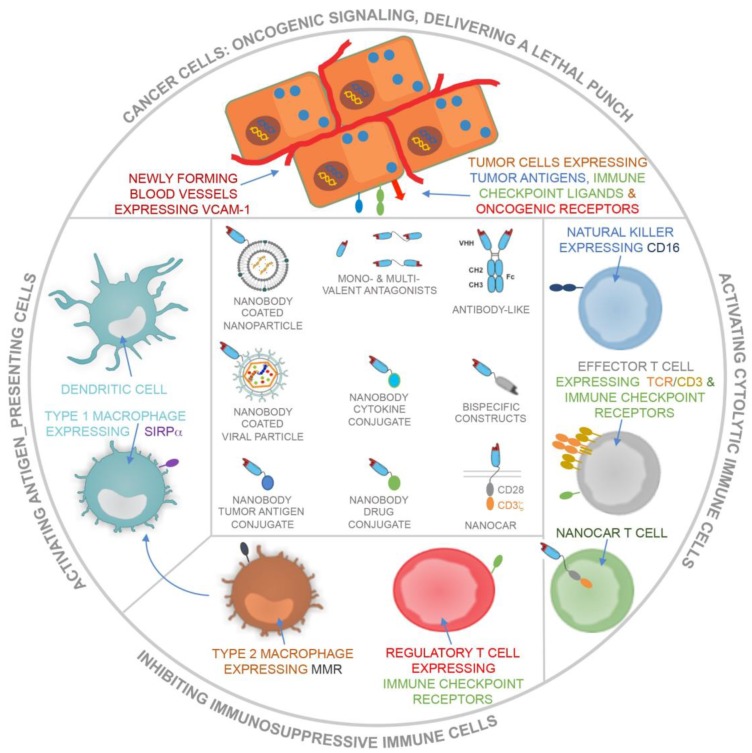
Schematic representation of the use of nanobodies and nanobody-derivatives for targeting of cancer cells and blood endothelial cells or for modulation of immune cells that can either activate (APCs, including DCs and type 1 macrophages), exert (cytolytic immune cells, including NK cells and CTLs) or suppress (type 2 macrophages and Tregs) antitumor immune responses.

Nanobodies have been studied extensively in the context of targeted cancer therapy and immunotherapy. Strategies embracing nanobodies in the fight against cancer can be categorized into the following types: the use of nanobodies to (1) dampen oncogenic signals, (2) deliver a lethal punch to cancer cells, (3) design cancer vaccines, (4) engage cytolytic cells and (5) prevent immunosuppressive events (Figure 4). Below we discuss the use of nanobodies according to these categories.

### Exploiting nanobodies to dampen oncogenic signals

Several types and subtypes of cancer are characterized by dysregulation of receptor tyrosine kinase (RTK) signaling, leading to an imbalance between cell growth, proliferation and cell death. Examples of RTKs that frequently have alterations in cancer cells, and therefore are constitutively providing oncogenic signals, are VEGFR, EGFR, HER2 and c-MET (hepatocyte growth factor receptor). Nanobodies that antagonize these RTKs have been developed. Additionally, nanobodies that bind VEGF and hepatocyte growth factor, the unique ligands of VEGFR and c-MET have been developed as well 121.

The versatility of nanobodies in the context of targeted therapy is exemplified by studies on targeting of EGFR and VEGF/VEGFR signaling. In the context of EGFR targeting, a biparatopic anti-EGFR nanobody fused to an anti-albumin nanobody, referred to as CONAN-1 was developed 122. CONAN-1 was reported to have a serum half-life of 2-3 days and to inhibit tumor outgrowth albeit at a lower potency as the EGFR targeting antibody cetuximab (Erbitux^®^). This lower potency may be related to a lack of Fc on the nanobody construct and suggests additional involvement of immune cells in eradication of EGFR-expressing tumors. Multi-specific nanobody formats have also been generated to target signaling mediated by VEGF and its receptor. A tri-specific nanobody targeting VEGF, angiopoietin-2 and serum albumin, has been co-developed by Ablynx^®^ and Boehringer Ingelheim. This construct, referred to as BI1836880, inhibits signaling mediated by VEGF and angiopoietin-2 and was shown in different *in vivo* models to be superior in efficacy in comparison to the antibody bevacizumab (Avastin^®^) 123. Inhibition of endothelial cell proliferation has also been shown with various monovalent nanobodies against different isoforms of VEGF, showing that the necessity to generate multivalent or multi-specific nanobody formats should be evaluated on a per case basis 124-126. A further improvement of efficacy of serum half-life extended antagonistic nanobodies can perhaps be augmented by arming these compounds with cytotoxic effectors, as discussed in the following paragraph.

### Exploiting cancer cell-specific nanobodies to deliver a lethal punch to cancer cells

Nanobodies that do not have antagonistic traits, yet target cancer cells, have been coupled to other technology platforms to deliver a targeted, lethal punch to cancer cells. Nanobodies have been coupled to (1) tumor necrosis factor-related apoptosis inducing ligand (TRAIL), a death inducing ligand 44,127, (2) a truncated form of the Pseudomonas exotoxin A 127-130, (3) various drugs and drug-loaded nanoparticles 131-138, (4) photosensitizers 139-143 and (5) therapeutic radionuclides 95,96,144-149. The reason to couple nanobodies to these toxic moieties is to bring them close to cancer cells, while minimizing toxic effects to healthy tissues, hence reducing potential adverse effects. Another strategy with a similar purpose is coupling of cancer cell-targeted nanobodies to enzymes for prodrug activation, ensuring drug activity only in the vicinity of cancer cells 150,151.

Targeting of DRs on cancer cells using TRAIL is an interesting strategy that was shown to act in concert with EGFR-targeting 152. Keeping this in mind, a bi-functional molecule consisting of an anti-EGFR nanobody coupled to TRAIL was generated 44,153. It was shown *in vitro* that the bi-functional molecule inhibited growth of several cancer cell types that did not respond well to EGFR blockade or DR engagement as a stand-alone treatment. Binding of EGFR by the nanobody induced DR clustering at the cancer cell membrane, thereby sensitizing these cells to TRAIL and downstream caspase-mediated apoptosis. Stem cells engineered to express the anti-EGFR nanobody-TRAIL (ENb-TRAIL) fusion protein were used *in vivo* in an orthotopic resection model of primary glioblastoma as a continuous source of the bi-functional molecule 44,153. ENb-TRAIL simultaneously binds to EGFR and DR5 receptor, present on tumor cells, leading to receptor clustering and the induction of apoptotic signals, which resulted in a significant increase in survival 44,153.

The truncated form of the Pseudomonas exotoxin (PE38) has been studied in conjunction with anti-VEGFR 128, anti-CD7 127,129 and anti-CD38 130 nanobodies. To generate so-called targeted toxins the nanobody in its monovalent or bivalent format is genetically coupled via a linker to the PE38 toxin. Both the VEGFR and anti-CD38 targeted immunotoxins were shown to compromise antigen-specific tumor cells *in vitro*, an effect that was also observed *in vivo* using CD7-targeted immunotoxins.

Among the drugs that are frequently used to treat various cancer types are cisplatin and its analogues, carboplatin and oxaliplatin as well as doxorubicin, RTK inhibitors and death effector molecules. As these drugs lack selectivity, nanobodies have been used to target them to cancer cells. In the case of delivery of platin-based chemotherapy two fusion proteins, termed NGC and NGCA, with different functional modules, were developed by *Huang et al* 131. The NGC fusion protein is composed of a biparatopic anti-EGFR nanobody for cancer cell targeting, a high-affinity gadolinium-binding domain for MRI imaging, and a C3-tag for drug conjugation. In addition, the NGCA fusion protein further contains an anti-albumin nanobody at the C-terminus of NGC, as this prolongs the *in vivo* circulation time. These nanobody containing fusion proteins were coupled to a maleimide-functionalized Pt(IV) prodrug (Mal-Pt), which was synthesized from cisplatin and coupled to the C3-tag. This theranostic drug delivery system enabled drug accumulation in EGFR^+^ tumors, which was most pronounced using NGCA, and delayed tumor growth with little toxicity when compared to classical treatment with cisplatin. Doxorubicin is another small molecule chemotherapy agent for which targeted delivery is of interest. This can be achieved by site-selective cysteine bioconjugation of a thioether propargyl carbamate linker bearing the anti-cancer drug to a nanobody against the HER2 antigen 132. Release of doxorubicin was achieved with palladium, resulting in decaging of propargyl carbamate protected lysine or tyrosine residues. It was shown that this reaction is suitable for drug delivery to cells. However, for *in vivo* applications the development of new palladium compounds is still needed. Another means of delivering doxorubicin is after its encapsulation in nanoparticles 154 such as liposomes 138, polymer-based nanoparticles and micelles 155, overall nanoparticles that could be coupled to nanobodies. Coupling of nanobodies to these nanoparticles often makes use of chemical reactive moieties that are present on the nanoparticle as a consequence of its PEGylation. The nanobody is coupled via cysteine chemistry or modified with N-succinimidyl-S-acetylthioacetate, as this does not affect the nanobody's binding capacity 85,156. Because this results in the presence of multiple nanobodies on the nanoparticle, a high avidity is obtained 157. The anti-EGFR nanobody has been used in view of nanoparticle targeting, guiding both the particle and exerting its antagonistic function. *Talleli et al.* studied targeted delivery of micelles 155 and in a follow-up study doxorubicin-loaded micelles 133. It was shown that these thermosensitive, biodegradable polymeric micelles themselves inhibit tumor growth *in vivo*, and that encapsulation of doxorubicin further increased the tumor inhibiting effects. Liposomes coated with anti-EGFR nanobodies, and loaded with the anti-IGFR-1R kinase inhibitor AG538 have been studied by *van der Meel et al.* to inhibit tumor growth 134. This is a particularly interesting approach as it has been shown that there is a crosstalk between EGFR and IGF-1R, which can lead to acquired resistance against EGFR targeted drugs 158. The latter is explained by the interaction of EGFR and IGF-1R, which can be directly through the association between their respective receptors and by inducing the availability of each other's ligands, or indirectly through the interaction of common binding partners or downstream signaling molecules. Subsequently, this provides a rationale to dual targeted therapies 158. Without AG538, the anti-EGFR coated liposomes induced downregulation of EGFR. Loading the liposomes with AG538 additionally affected IGF-1R signaling and further increased the inhibitory effect on cancer cell growth *in vitro* 134. *In vivo*, growth inhibition was observed in one of the two cell lines examined in a mouse xenograft model 135. Also nanobody-coated polymers have been studied for delivery of drugs to cancer cells. Polymers decorated with anti-HER2 136 or anti-MUC1 nanobodies 137 were shown to enable selective cancer cell targeting. Encapsulation of plasmid DNA and hence delivery of genetic material was achieved in this way. More specifically, MUC1-targeted delivery of plasmid DNA encoding for truncated-Bid (under the control of a MUC1 promotor) was shown to result in expression of the transgene, resulting in considerable cell death 137.

Instead of delivering a drug, cancer cell-specific nanobodies have also been studied to deliver enzymes that cleave prodrugs into their active form, as such ensuring minimal off-target drug activity. An example hereof is the fusion of the enzyme β-lactamase from *E. cloacae* P99 to a high-affinity anti-CEA nanobody. The chosen β-lactamase enzyme efficiently converted the prodrug 7-(4-carboxybutanamido) cephalosporin mustard in phenyl-enediamine mustard at the surface of CEA-expressing colon cancer cells *in vitro*, thereby inducing cytotoxicity. This nanobody-enzyme conjugate induced tumor regression and in some cases even cured mice of established tumor xenografts 150. Another example is the immunoconjugate designated L-DOS47, which is comprised of a nanobody targeting CEACAM6 coupled to an urease enzyme that converts urea into ammonia *in situ* and as such induces toxicity. The specificity and activity of L-DOS47 was shown and this nanobody-conjugate is being explored in a phase I/II clinical trial for non-small cell lung cancer 151.

Hitting a photosensitizer with light of a particular wavelength in an oxygenated environment results in formation of reactive oxygen species (ROS). These ROS in turn damage proteins, lipids and/or nucleic acids, and as such induce cell death. Monovalent (7D12) and biparatopic (7D12-9G8) nanobodies targeting EGFR have been used to selectively deliver the photosensitizer IRDye700DX, a fluorescent dye that also allows OI. *In vitro* studies showed that these anti-EGFR-IRDye700DX conjugates specifically induced cell death of EGFR overexpressing cells in low nanomolar concentrations, while IRDye700DX alone or anti-EGFR-IRDye700DX conjugates in the absence of light did not induce cell death 140. Internalization of IRDye700DX was observed when the photosensitizer was coupled to the biparatopic format of the anti-EGFR nanobody, which resulted in a more pronounced induction of cell death 140. However, enhanced cytotoxicity upon internalization of nanobody photosensitizer conjugates was contradicted in a study by *van Lith et al.* 141. Herein, the anti-EGFR nanobody was conjugated to a cell-penetrating peptide (CPP) to enhance internalization of the IRDye700DX photosensitizer. This discrepancy is likely explained by the biparatopic format of the anti-EGFR nanobody used by *Heukers et al.,* which might induce EGFR downregulation and as a consequence reduced oncogenic signaling 140, much in the same way as observed when anti-EGFR nanobodies are coupled to nanoparticles 134,155. Induction of selective tumor cell death was also studied *in vivo* in immunodeficient mice growing orthotopic tongue tumors, showing that both 7D12 and 7D12-9G8 anti-EGFR-IRDye700DX conjugates allowed selective OI of the tumors, and that in particular the 7D12 anti-EGFR-IRDye700DX conjugate consistently induced near-complete tumor eradication with little to no toxicity in healthy tissues 139. The potency of this strategy warrants further studies to translate this technology from bench to bedside.

Similar as photosensitizers, branched gold nanoparticles kill cancer cells when excited by NIR-light, but by generating heat instead of ROS. *Van de Broek et al*. and *d'Hollander et al*. site-directionally conjugated such gold nanostars to anti-HER2 nanobodies and showed *in vitro* that they precisely killed HER2-expressing cancer cells when triggered by laser-light 142. Moreover, *in vivo* these HER2 nanobodies coupled to gold nanostars were able to reach HER2^+^ xenografted tumors 143.

Targeted radionuclide therapy (TRT) is a systemic treatment with radioactively labeled cancer-specific probes purposed to selectively hit diseased cells. As radioactive labels α- or β^-^-emitters are used, which release their energy in the proximity of the cancer cells, thereby causing irreparable DNA damage. The potential of nanobodies as targeting moieties for TRT in preclinical cancer models has been extensively investigated using the therapeutic β^-^-particle emitting radionuclides Lutetium-177 and Iodine-131, and more recently the α-emitting radionuclides Astatine-211, Actinium-225 and Bismuth-213 95,96,144,145. Early proof-of-concept studies using β^-^-particle emitting radionuclides coupled to nanobodies showed delivery of lethal radiation doses to developing tumors with a negligible level of irradiation to healthy tissues, except for kidneys, which were the dose-limiting organs. However, a generic method to reduce kidney retention of radiolabeled nanobodies was described, in particular the use of untagged nanobodies with co-infusion of the plasma expander Gelofusin 95. Preclinical studies on β^-^-TRT have been performed in several cancer models, including multiple myeloma 144, breast and ovarian cancer 95,145, and non-Hodgkin lymphoma 96, using nanobodies targeting the 5T2MM paraprotein, HER2 and CD20, respectively. In these studies, nanobody-based β^-^-TRT led to a significant blockade of tumor growth and as a consequence a significant difference in event-free survival between the treated and the control groups. However, repeated dosing was required to achieve an effective therapeutic dose in tumors, which is in part explained by the low linear energy transfer (LET) of β^-^-particles (LET: 0.1-1 keV/µm) and the rapid clearance of nanobodies. Because α-particles have a higher LET (50-100 keV/µm) with a short penetrating range (40-80 µm), α-TRT has been proposed as an attractive strategy to eradicate residual cancer cells 146,147. Several research groups have reported on the characterization of HER2- and PSMA-targeting nanobodies labeled with the α-emitting radionuclides Astatine-211, Actinium-225 and Bismuth-213 147-149. In these studies specific uptake of the radiolabeled nanobodies was shown with high tumor-to-normal organ ratios (except for kidneys). Moreover, it was shown using Bismuth-213 coupled to anti-PSMA nanobodies that DNA double-strand breaks were induced *in vivo* in tumor cells 149. Taken together, these studies support the further development of nanobody-mediated TRT.

### Exploiting nanobodies to design cancer vaccines targeted to antigen-presenting cells

The goal of cancer vaccination is to activate CTLs. These can selectively recognize and kill cancer cells irrespective of their location, and can form an immunological memory, ready to act when cancer cells with the same properties pop up. Activation of CTLs requires tumor antigen presentation in MHC-I molecules and co-stimulation by professional APCs of which DCs have been extensively studied for cancer vaccination 56,159.

Adoptive transfer of autologous DCs manipulated *in vitro* to present tumor antigens has proven successful in several clinical trials 160. However, to circumvent the laborious and expensive procedure of generating *ex vivo* DCs, strategies have been developed to manipulate DCs *in situ.* Nanobodies have been coupled to several technology platforms designed to deliver tumor antigens or potentiate the APC's antigen-presenting capacity. It is contended that targeting of APCs will enhance the vaccine efficacy and will reduce potential adverse effects.

Nanobodies have been generated against multiple proteins that are expressed on the surface of APCs, including CD11b, CD36, MHC-II 161, CD1d 162, PD-L2 163 and Clec9a 164. Moreover several nanobodies that target APCs, however for which the antigen has not yet been identified, have been described, including nanobody R3_13 47, DC1.8 and DC2.1 119. *In vivo* biodistribution studies suggest that DC1.8 targets conventional DCs (cDCs) of murine origin, while DC2.1 targets myeloid APCs, including cDCs, plasmacytoid DCs and macrophages. These nanobodies were studied in the context of virus-based cancer vaccines, based on adenoviruses 165 and lentiviruses 47,49,55,57,166. Incorporation of the nanobody into lentiviral vectors is achieved using the nanobody display technology, which exploits the budding mechanism of lentiviral vectors to incorporate the nanobody together with a binding-defective, fusion-competent glycoprotein in the viral envelope 49,167. This strategy allows high titer production of lentiviral vectors coated with nanobodies. When using DC1.8 and DC2.1, selective, nanobody-dependent infection of mouse DCs and macrophages both *in vitro* and *in situ* was shown. Moreover, this strategy was translated to a human model, showing selective infection of *in vitro* generated or lymph node-derived DCs and macrophages 47. An adenovirus-based vaccine was also successfully redirected to DCs by replacement of the adenoviral fiber knob with fiber-fibritin chimeras fused to DC1.8 165. Against the expectation, the APC-targeted lentiviral cancer vaccine was less potent in activation of CTLs than non-targeted lentiviral cancer vaccines 57. This was explained by (1) a strong induction of type I interferon (IFN), which is a result of recognition of viral components by the APCs and hampered the translation of the delivered tumor antigen; and (2) lack of stromal cell transduction with reduced MHC-I mediated antigen presentation 168. In contrast, enhanced immunogenicity was observed for peptide-based vaccines when these were coupled to nanobodies specific for MHC-II, CD11b or CD36 161. It was observed that coupling of peptides to the nanobody targeted to MHC-II elicited strong activation of CD4^+^ T cells, which support APCs and CD8^+^ T cells in their function. The strongest activation of CD8^+^ T cells was observed when using the anti-CD11b nanobody for targeting 161. These studies suggest that nanobodies are indeed suited to target tumor antigens to APCs, however point out that a smart choice of a technology platform is required to ensure that the APC reaches its full potential to present the tumor antigens.

DCs can acquire tumor antigens in tumors. However tumor-derived factors render these DCs unable to activate CD4^+^ and CD8^+^ T cells. Therefore, strategies have been explored to activate these DCs, of which delivery of cytokines is an example 166. As systemic delivery of cytokines is often correlated to toxicity, nanobodies have been used to engineer 'Activity-on-Target cytokines' or AcTakines. These mutant cytokines display a reduced receptor-binding affinity, however upon fusion to nanobodies their activity is restored on the nanobody bound cell populations. In the case of IFN or 'AcTaferon', this hypothesis was confirmed with a nanobody specific for programmed death-ligand 2 (PD-L2) on APCs as it appeared up to 1,000-fold more potent on target cells, allowing specific signaling in selected cell types only 163. A strong antitumor activity was also observed when a nanobody against the cDC1 marker Clec9A was used as adapter in murine melanoma, breast carcinoma and lymphoma models and against human lymphoma in humanized mice without any detectable toxic side effects 164,169.

While the strategies above use nanobodies to deliver tumor antigens or cytokines to APCs, agonistic nanobodies that target CD1d have been used 'as such' to potentiate DCs 162. CD1d is an antigen-presenting molecule involved in the presentation of glycolipids to NKT cells, which are recognized as major contributors to antitumor immunity 170. Similar to CD4^+^ T cells, NKT cells support APCs and CTLs in their activity, and can exert direct cytolytic effects on cancer cells. *Lameris et al.* generated anti-CD1d nanobodies and showed that 2 out of 22 nanobodies had the capacity to induce NKT cell-independent production of interleukin (IL) 12 by monocyte-derived DCs, thereby supporting the DC's ability to activate antitumor immunity. This study shows that nanobodies can be used as such to potentiate the APC's stimulatory capacity, provided that the target is well chosen. Other potential targets for agonistic nanobodies include co-stimulatory molecules that signal towards APCs, such as CD40 171 and CD83 172.

### Exploiting nanobodies to engage cytolytic cells

Cancer vaccination is one approach in which nanobodies are studied to activate cytolytic immune cells to recognize and kill cancer cells. This strategy however requires targeting of APCs. Alternative approaches using nanobodies have been designed that act directly on cytolytic immune cells, thereby bypassing the targeting of APCs.

A multitude of cytokines acts as signals for T cell activation, proliferation and survival. To target these signals to T cells within the TME, and therefore avoid overwhelming systemic T cell activation, cytokines have been coupled to nanobodies targeting antigens highly expressed in the TME. An anti-CEA nanobody-Fc fusion was further coupled to IL-15 linked to its soluble receptor IL15-R to increase its function, and this construct was used in a xenograft model, showing strong antitumor effects associated with CD8^+^ T cell recruitment 173. Also IL-2 and IFN-γ have been coupled to nanobodies, in this case targeting PD-L1. It was shown that the anti-PD-L1 nanobodies delivered the cytokines in the TME using a melanoma as well as pancreatic cancer model. Combining the nanobody-mediated delivery of IL-2 and IFN-γ with tumor-specific antibody therapy resulted in tumor growth inhibition, which coincided with increased CD8^+^ T cell numbers. Delivery of IL-2 however also increased regulatory T cell numbers, warranting the use of a mutated form of IL-2 with affinity for binding to the IL-2 receptor on CD8^+^ T cells. Next to increasing CD8^+^ T cell numbers, delivery of IFN-γ acted on myeloid cells, redirecting them towards an MHC-II^+^ phenotype, which is correlated to an antitumor function 174.

While cytokines provide important signals during the activation of CD8^+^ T cells, they only act as co-stimuli. Without engagement of the TCR and subsequent CD3ζ signaling, CD8^+^ T cells cannot differentiate into CTLs. Therefore, strategies have been developed to mimic CD3ζ signaling in the absence of MHC-I:peptide mediated TCR triggering. These rely on binding of CD3 by agonistic antibodies or antibody fragments that are cross-linked. Nanobodies that bind EGFR on cancer cells have been coupled in their monovalent format or as a trimer to anti-CD3 scFvs to ensure cross-linking and as such T cell activation 58,59. Such constructs are referred to as LiTEs or bispecific light T cell engagers, and were shown to retain high tissue penetration capacity and to enable strong levels of T cell activation. To further increase the T cell activating capacity of LiTEs, nanobodies binding 4-1BB, a co-stimulatory receptor on T cells, have been incorporated in the construct, showing accumulation in the TME and efficient activation of antitumor responses 175. Activation of γδ T cells, in particular Vγ9Vδ2 T cells that exert antitumor activity, has been achieved using a similar principle. Activation of TCR signaling without functional recognition of MHC-peptide complexes was achieved using agonistic nanobodies that bind the Vγ9Vδ2 TCR and that were coupled to anti-EGFR nanobodies to ensure cross-linking. When combined with Vγ9Vδ2 T cells this construct enhanced the cytolytic response of the Vγ9Vδ2 T cells against EGFR^+^ cancer cell lines *in vitro* as well as in xenografted mice 176.

To ensure on-target cytolytic activity of CD8^+^ T cells, CAR T cells have been developed. These are patient-derived T cells that are genetically modified to express a CAR that combines T cell cytotoxicity with the MHC-independent antigen recognition of antibodies. The concept was first introduced by *Gross et al.,* who cloned V_H_ and V_L_ parts of an antibody to the intracellular parts of TCRα and TCRβ in two different genes 177. This design was then further adjusted by the same group, when they assembled one single genetic construct, consisting of a scFv, coupled to intracellular FcRγ or CD3ζ in 1993 178. This set the basis for further CAR design and adjustment over the years, which is nowadays conventionally build-up of a single gene encoding an antigen recognizing part (conventionally a scFv), coupled to a spacer (derived from CD8α, CD28, IgG1 or IgG4), a transmembrane domain (derived from CD3ζ, CD4, CD8α or CD28) and to intracellular co-stimulatory (CD28, 4-1BB, OX40, ICOS and/or CD27 derived) and activation (usually CD3ζ) domains 179. While most research has been focused on the optimization of the intracellular signaling domain, fewer efforts have been made in the optimization of the format of the extracellular, antigen binding part of the CAR 180. ScFvs derived from clinically approved or tested antibodies are used most commonly because their behavior and target-specificity in patients is well characterized. However, some drawbacks are attributed to this antigen-binding format in a CAR context. For instance, scFvs can be immunogenic and instable. This has been proven to limit clinical efficacy because of an anti-CAR immune response, and to lead to premature T cell exhaustion 181-183. The latter was found to be due to the vulnerability of scFv framework regions to aggregation, which leads to antigen-independent T cell activation, shortly followed by premature exhaustion, limiting CAR T cell persistence *in vivo* 184,185. Consequently, several groups have successfully replaced the scFv with a nanobody in a CAR context, to create nanoCARs. These nanoCARs target different tumor cell-associated markers, such as PSMA 52 and MUC1 51 for solid tumors and CD38 for myeloma 53. Other groups have developed nanoCARs directed against markers of tumor stroma, such as PD-L1, EIIIB fibronectin splice variant and VEGFR2 54,186. Although difficult to compare directly with scFv-based CARs because of differences in protocols and constructs, these studies show that nanoCARs make valid alternatives for classical CARs and might open the door to a more efficient CAR design.

The concept of universal CARs (uniCARs), which are CAR T cells recognizing a molecule or peptide that is coupled to a cancer-targeting module (TM), administered separately, has also been introduced with nanobodies 187. In this pre-targeting approach the TM will guide uniCARs to the tumor target in a concentration-dependent manner. In the absence of a TM, the uniCAR T cells are inactive. This allows the repeated stop-and-go retargeting of tumor cells 188*.* Recently also, nanobody-based CAR NK cells have been validated in xenografts of T cell acute lymphoblastic leukemia 189, opening doors to off-the shelf products.

Altogether, several recent studies show how nanobodies are slowly finding their way in CAR cell therapy, with different side tracks being explored. Though most studies using nanoCARs are still in the early stages, few examples prove that nanoCARs are paving the way to clinical applications. The CAR T cell therapy from Nanjing Legend Biotech^®^ uses two BCMA-specific nanobodies in tandem, targeting myeloma cells and is proving to be effective in phase I/II clinical trials (NCT03758417) 190,191. More recently, a phase I trial with CD19/CD20 bispecific nanoCAR T cell therapy for B cell lymphoma was initiated (NCT03881761). Results from these trials will prove whether nanobody-based CAR T cell therapy can add value in the field of adoptive T cell transfer, at points where scFvs fall short.

Next to CD8^+^ T cells, NK cells, which are innate cytolytic immune cells, can be harnessed in the fight against cancer. They can exert direct cytolytic functions or can recruit DCs and aid T cell activation through cytokine and chemokine production. Modification of NK cells with nanoCARs is one way to exploit the antitumor properties of NK cells 189. Another strategy that has been explored is the development of BiKEs or bispecific killer cell engagers. Herein the ability of NK cells to exert cytolytic functions when the receptor CD16 (FcRIII) is bound is used. As coupling of cancer-specific nanobodies to Fc fragments can impact on the nanobodies' properties, as excellently reviewed by Saunders KO 192 describing the multifaceted functions of Fc, a nanobody that binds CD16 with high affinity has been generated, designated "C21" 62. Multimerization of this nanobody allowed activation of NK cells, as shown by the production of IL-2 and IFN-γ 62. Coupling of this nanobody to an anti-MUC-1 61 or an anti-CEA 193,194 nanobody was shown to mediate tumor growth inhibition when injected on a daily basis in mice receiving peripheral blood mononuclear cells and xenografted with MUC-1^+^ or CEA^+^ colon carcinoma tumors. Comparison of trastuzumab (Herceptin^®^) to a construct in which nanobody "C21" was coupled to a bsFab build from anti-HER2 nanobodies showed comparable efficacy *in vivo* on cells that are highly HER2^+^, such as SK-BR-3 and BT474, however superior activity of the nanobody-based construct when HER2 expression was low (MCF-7 model) 195.

### Exploiting nanobodies to revert immunosuppressive events

Tumor cells exploit different suppressive pathways and cells to avoid antitumor immune responses, allowing them to continue their growth undisturbed and eventually to metastasize. In this paragraph we address the following immunosuppressive mechanisms, (1) inhibitory ICPs, (2) immunosuppressive cytokines and (3) myeloid cells like M2-macrophages, against which nanobodies have been developed.

The discovery of the ICPs CTLA-4 and PD-L1 led to the award of the Nobel prize for medicine to James Allison and Tasuku Honjo in 2018 and impacted the current therapeutic landscape for patients with solid and hematological tumor malignancies 196. Regarding the ICP CTLA-4, efforts have been taken to develop nanobodies for therapeutic applications. In the study of *Wan R et al.*, the treatment of melanoma (B16) bearing mice with mouse CTLA-4 targeting nanobody "NB16" significantly delayed their tumor growth and survival time 197. In contrast, *Ingram JR et al.* demonstrated that nanobody-mediated blocking of mouse CTLA-4 only induced minimal effects on antitumor responses 107. Interestingly, the lack in therapeutic efficacy was related to the lack of an Fc portion on nanobodies. Fusion of nanobody "H11" to a murine IgG2a constant region could dramatically enhance its antitumor effect. These studies again highlight that the necessity to modulate the nanobody format should be evaluated on a per case basis. Therapeutic nanobodies have also been developed against the ICP PD-L1 in order to counteract immune inhibition through the ligation with its receptor PD-1 that is highly expressed on activated T cells 111,198-200. Nanobody mediated blockade of PD-L1, present on DCs, could significantly increase the activity of T cells upon antigen presentation, an effect that was not seen with the FDA-approved anti-PD-L1 antibody Avelumab 198. Moreover, the study of *Zhang et al.* demonstrated *in vivo* therapeutic effects of their PD-L1 targeting nanobody when fused to a human IgG1 Fc 199. This nanobody, designated as KN035, is currently being evaluated in 2 different clinical trials for the treatment of patients with advanced solid tumors (NCT03101488, NCT03248843).

Another ICP ligand is CD47. CD47 is expressed in a wide range of malignancies and negatively regulates phagocytosis through the interaction with its receptor SIRP1α, expressed on macrophages. A study using a mouse melanoma model has shown that blockade of the CD47-SIRP1α interaction using nanobody "A4" was only able to induce tumor rejection responses when locally delivered and combined with antitumor antigen antibodies, PD-L1 blockade and/or GVAX vaccination 201. In contrast with the Fc fused CTLA-4 blocking nanobodies mentioned above, fusion of "A4" to a constant region did not improve its therapeutic efficacy and even induced anemia since CD47 is also expressed by red blood cells.

The cytokine tumor necrosis factor alpha (TNF-α) works immunosuppressive by increasing the expansion of immunosuppressive myeloid cells and regulatory T cells at the tumor site 202,203. Nanobodies targeting TNF-α were able to reduce lung metastasis *in vivo* and worked synergic with the antimitotic agent paclitaxel 204.

Several strategies have been explored to directly target immunosuppressive myeloid cells like for example macrophages. The drugs gefitinib and simvastatin are known to have an effect on the activation of tumor-associated macrophages 205,206. Liposome coupled, PD-L1 targeting nanobodies were loaded with these drugs and showed a potent antitumor effect when administered *in vivo* 207*.* A switch in M1/M2 ratio in the treated mice in favor of M1-macrophages was observed. Moreover, nanobodies targeting MMR, present on pro-tumor M2-macrophages, have been generated and used in therapeutic settings when fused to other proteins and nanoparticles. Coupling of the MMR specific nanobody to pro-apoptotic proteins like second-mitochondria derived activator of caspases showed specific targeting to and modulation of M2-macrophages 208. Moreover, *Nuhn et al.* conjugated anti-MMR nanobodies to polymeric maleimide-functionalized nanogels and showed effective nanoparticle delivery to MMR-expressing macrophages *in vitro* and *in vivo* when administered in tumor bearing mice 209. Consequently, they observed a significant reduction of M2-macrophages in the TME, which could pave the road for targeted eradication, or modulation of immunosuppressive cells.

## Theranostics in immuno-oncology using nanobodies and nanobody derivatives

The focus for the treatment of most cancers is evolving to more expensive therapies where predictive markers are not only clinically relevant but also an economic requirement. Diagnostic tests like immunohistochemistry are current practice but are unable to portray whole tumor expression levels and this is even worse for metastatic lesions. This could explain the failure to accurately predict outcome responses in all patients. Whole body, noninvasive imaging modalities such as PET, SPECT, MRI and OI, using nanobody-based tracers, could fulfill these shortcomings and could be implemented repetitively without the need of collecting invasive biopsies.

Until now, we described the use of nanobodies in the context of immuno-oncology for either imaging or therapeutic applications. We are convinced that many of the described nanobodies hold the potential to be used as molecular imaging probes as well as therapeutic agents. The term “theranostic” was initially put forward to describe the development of diagnostic tests alongside the application of a therapy targeted towards a specific molecular feature, as exemplified by Herceptin^®^ and HercepTest^®^, which were simultaneously approved by the FDA in 1998 for the treatment and diagnosis of Her2 expressing breast cancers 210. Hence, historically, the theranostics paradigm refers to different methodologies and compounds that unite diagnosis and therapy of the same molecular pathways and disease indication in a practice that is also called precision medicine. Currently, the term theranostics is used in a much stricter sense and rather refers to agents that are identical or closely related and that harbor the potential to be used both for diagnostic as well as for therapeutic purposes. In this paragraph we will elaborate on nanobody-based compounds for use as true theranostics. A summary of published examples is made in Table 1.

First of all, nanobodies targeting cancer-specific membrane proteins (e.g. HER2, EGFR, M-protein, CD20 and CD38) have been evaluated for both imaging and therapeutic applications 73,95,96,122,130,144,147,211. The clearest example of nanobody theranostics is where both diagnostic tracers and therapeutic compounds are radiolabeled, in a TRT approach 96,144. The radiolabel can be different, e.g. Gallium-68 or Fluor-18 for PET imaging and Actinium-225 for α-TRT. But sometimes the radiolabel is the same such as Iodine-131 labeled nanobodies that are first used at low doses in SPECT imaging for diagnosis and dose estimations, and then at higher doses for TRT 145. Of importance is that the diagnostic and therapeutic nanobody-radiopharmaceuticals have similar pharmacokinetics and biodistribution profile.

Nuclear imaging with nanobodies could be a guide for other nanobody-based therapeutic modalities as well. Examples of these are nanobody-toxin fusions 18,127,128 or nanoCAR T cells 50,52,53. In these settings PET imaging is the dominant diagnostic modality due to its superior sensitivity, resolution and quantifiability. In the nanoCAR T application PET could be useful to predict homing of the re-targeted T cells to the tumor.

Another clear example of nanobody-theranostics is OI and photodynamic therapy 121. Here the cancer-specificity of NIR-labeled nanobodies is first exploited to visualize the tumors (diagnosis) and help the surgeon to better delineate tumor boundaries (therapy) 212. After the image-guided surgery, residual cells can then be killed by activating the nanobody-photosensitizer-conjugates on the membrane of the targeted cells by light.

Next, nanobodies targeting specific immune cells subsets like DCs can be used to detect their presence in mm-sized tumors using different imaging modalities 119. The same nanobodies can then be used to target peptide vaccines or cytokines to these cells and elicit immunogenicity responses 49,213-215.

Finally, nanobodies have been generated against ICPs due to their important role in dampening antitumor immune responses. For example, CTLA-4 or PD-L1 targeting nanobodies, when labeled with radioisotopes, can be used to noninvasively detect using PET or SPECT imaging which type of immunosuppressive pathway dominates the TME 109,111,112,197. Then, these nanobodies when administered as such, or combined with chemokines/cytokines or implemented in nanoCARs can restore the antitumor immune responses *in vivo* 54,107,174,198,199,216.

## Outlook: evolution from bench to bedside

Since their first observation, over a quarter century ago, many nanobody-based pharmaceuticals, after a preclinical phase, are now in or on the path towards clinical testing in the area of oncology. Here the nanobodies block essential protein-protein interactions, re-target effector cells or act as nanobody-drug conjugates as well as tracers for molecular imaging. The extraordinary features of nanobodies - small size, stability, affinity, easy of engineering in multifunctional constructs - will continue to fulfill a central role in future scientific research in the field of antibody engineering. Preclinical and clinical data such as those with HER2 targeting nanobodies support the claims of the great potential of labeled nanobodies as diagnostic agents. Moreover, increasing pre-clinical studies have evaluated the use of nanobodies to successfully combat a variety of different tumor models *in vivo*. This report contains a state-of-the-art overview of the use of nanobodies as therapeutic and diagnostic agents in the field of immuno-oncology and their promise as theranostics in the context of cancer. Their small size makes them ideally suited for noninvasive imaging of malignant cells or processes linked to malignancy such as angiogenesis and immunosuppression. Consequently, the potential of visualizing these markers will act as a modality to guide and monitor treatments of patients. Furthermore, nanobodies are more and more studied in therapy applications. Despite the lack of any Fc-mediated effector functions, pre-clinical studies hint towards a substantial value in applications such as the blockade of receptor signaling present on tumor cells and/or immune cells and targeted delivery of various agents ranging from toxins, pro-apoptotic proteins, radionuclides to cytokines. Preclinical studies on the applications of nanobodies in immuno-oncology are vast, and translation of some of these methodologies in a clinical setting is either already happening or a possibility in the near future.

## Figures and Tables

**Table 1:**
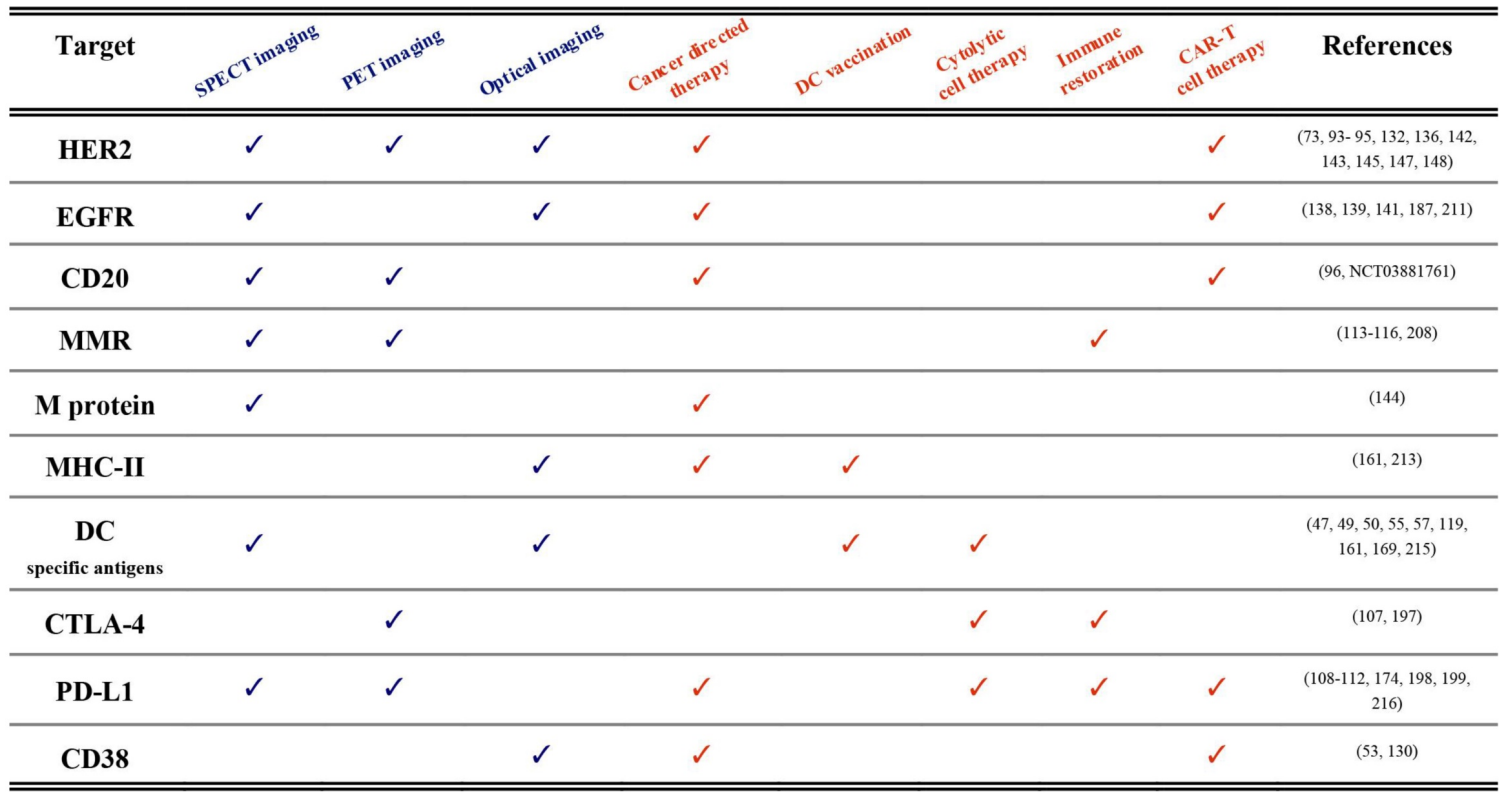
Published theranostic applications (pre-clinical and clinical) of nanobodies in immuno-oncology. Diagnostic technologies are labeled blue, whereas therapeutic approaches are depicted in orange. Examples and references are shown per targeted antigen.
